# Therapeutic Implication of SOCS1 Modulation in the Treatment of Autoimmunity and Cancer

**DOI:** 10.3389/fphar.2019.00324

**Published:** 2019-04-11

**Authors:** Jatin Sharma, Joseph Larkin

**Affiliations:** Department of Microbiology and Cell Science, Institute of Food and Agricultural Sciences, University of Florida, Gainesville, FL, United States

**Keywords:** autoimmunity, SOCS1, JAK/STAT, jakinibs, cancer, cytokines

## Abstract

The suppressor of cytokine signaling (SOCS) family of intracellular proteins has a vital role in the regulation of the immune system and resolution of inflammatory cascades. SOCS1, also called STAT-induced STAT inhibitor (SSI) or JAK-binding protein (JAB), is a member of the SOCS family with actions ranging from immune modulation to cell cycle regulation. Knockout of SOCS1 leads to perinatal lethality in mice and increased vulnerability to cancer, while several SNPs associated with the SOCS1 gene have been implicated in human inflammation-mediated diseases. In this review, we describe the mechanism of action of SOCS1 and its potential therapeutic role in the prevention and treatment of autoimmunity and cancer. We also provide a brief outline of the other JAK inhibitors, both FDA-approved and under investigation.

## Introduction

Cytokines are small glycoproteins secreted by a variety of immune and non-immune cells. These molecules govern a range of processes including, but not limited to, hematopoiesis, inflammation, cell proliferation, survival, apoptosis, and chemotaxis. As such, intricate modulation of cytokine signaling is required for maintaining immune system homeostasis and regulation of inflammatory responses. Cytokine receptors typically belong to one of the following families: IL-1 receptor superfamily, TNF-receptor family, IL-17 receptor superfamily, G-protein coupled receptor (GPCR) superfamily, transforming growth factor superfamily, receptor tyrosine kinase superfamily (RTK), and type I and II cytokine receptor superfamily. Among these, only type I and II cytokine receptors are physically associated with JAKs (Gadina et al., 2001). JAKs are tyrosine kinases whose primary targets are Signal transduction and activator of transcription (STAT) proteins. JAK-STAT signaling is the canonical pathway induced by cytokines binding to type I or II cytokine receptors, though other major pathways such as PI3K/AKT and MAPK (p38, JNK, and ERK1/2) are also involved either directly or indirectly depending on the cytokine and the target cell type. Cytokine signaling can be regulated in the following ways - (1) by modulation of cytokine gene expression, (2) regulation at the receptor level or (3) at the stage of signal transduction. Cytokine receptor modulation can occur through changes in expression of the cytokine receptor, blockade of receptors via natural antagonists/decoy ligands (e.g., IL-1Ra blocks IL-1R) (Seckinger et al., 1987), or blockade of ligand via decoy receptors (e.g., sgp130 blocks sIL-6R signaling) (Jostock et al., 2001). In terms of signal transduction, changes in the expression of signal transducing elements, or their respective regulators, may serve to modulate the signal. Signal transduction regulation may occur as either post-transcriptional or post-translational regulation. While regulation at the ligand or the receptor level is more specific, modulation at the signal transduction stage allows control over multiple cytokine signals at once. The JAK family of non-receptor tyrosine kinases comprises of four members: JAK1, JAK2, JAK3, and TYK2. Their canonical targets are the STAT family proteins which includes STAT1, STAT2, STAT3, STAT4, STAT5a, STAT5b, and STAT6. The STAT proteins are transiently active when phosphorylated by JAKs. Phosphorylated STATs form homo/heterodimers to act as transcription factors, though unphosphorylated STAT dimers may be present and exert biological activity (Braunstein et al., 2003; Yang and Stark, 2008; Sgrignani et al., 2015; Butturini et al., 2016). JAK/STAT signaling cascades, under normal conditions, are regulated by Protein inhibitor of activated STAT (PIAS), phosphatases such as SHP-1 and SHP-2, and the members of the SOCS family of proteins (Chung et al., 1997; Naka et al., 1997; Liu et al., 1998; Barber, 2001; Kirito, 2002; Kelley, 2008). Recent reports have elucidated that JAK1/2 may also be able to activate the PI3K-AKT pathway via phosphorylation of p85 (the regulatory subunit of the PI3K enzyme) (Yamada et al., 2012). Hence, the regulators of the JAK family indirectly modulate PI3K signaling as well.

**FIGURE 1:**
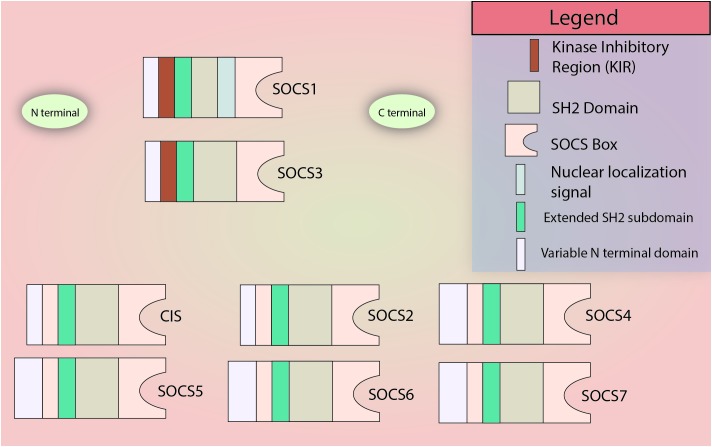
SOCS family members. All members of the SOCS family contain a variable N terminal domain, an SH2 domain, an extended SH2 subdomain (ESS), and the C-terminal SOCS Box domain. The N-terminal KIR domain is restricted to SOCS1 and SOCS3. Only SOCS1 is known to contain a nuclear localization signal. Please note: In most SOCS proteins, there is a little C-terminal sequence left after the SOCS Box which has not been illustrated in the figure for simplicity.

**FIGURE 2:**
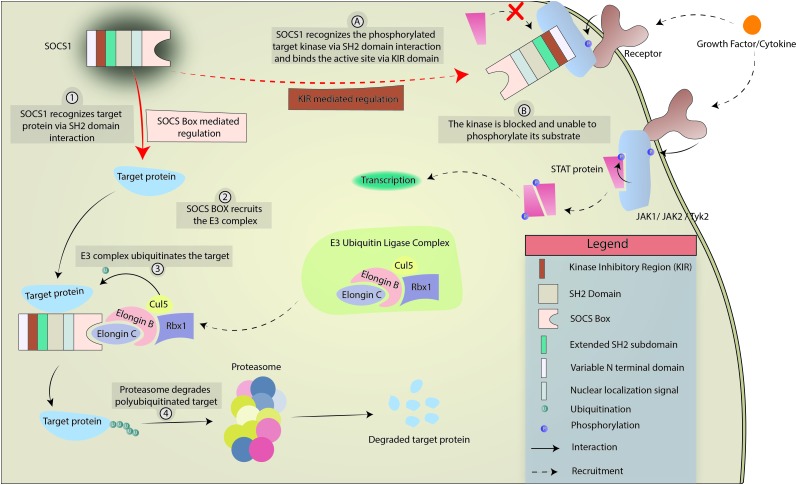
Mechanism of SOCS1-mediated regulation of cytokine and growth factor signaling. SOCS1 regulates intracellular processes in 2 ways, limned as either numerical (SOCS box-mediated) or alphabetical (KIR-mediated). In SOCS Box mediated regulation, SOCS1 interacts with target protein via SH2 domain interaction and uses the SOCS Box to recruit the E3 ubiquitin ligase complex. The E3 complex polyubiquitinates the target which is eventually degraded by the proteasome. In KIR-mediated regulation, SOCS1 interacts with a target kinase (JAK1, JAK2, or TYK2) via SH2 domain interaction. The KIR acts as a pseudosubstrate and blocks the phosphorylation site of the kinase, preventing the kinase from phosphorylating its target.

The SOCS family consists of a group of 8 intracellular proteins: SOCS 1–7 and CIS, the first member to be discovered, (Barber, 2001; Krebs and Hilton, 2001) all possessing an SH2 domain, C-terminal SOCS box, N-terminal extended SH2 subdomain (ESS), and a variable N-terminal region (see Figure 1). Additionally, SOCS1 and SOCS3 also possess a KIR. The SH2 domain imparts specificity to the protein by binding to specific phosphotyrosine residues on the target (Koch et al., 1991; Liau et al., 2017), allowing the KIR domain to inhibit kinase activity by acting as a pseudosubstrate, in case of SOCS1 and SOCS3. The SOCS box can recruit factors to form E3 ligase complex that tags the target protein for ubiquitination, leading to its proteasomal degradation (Zhang et al., 1999; Bullock et al., 2007; Liau et al., 2018; Figure 2 demonstrates the mechanism briefly). Notably, only SOCS1 has been reported to have a nuclear localization signal (NLS) (Baetz et al., 2008). The SOCS1 NLS is known to enable p65 destabilization in the nucleus and it was shown in mouse CD11c^+^ cells that SOCS1ΔNLS has impaired ability to inhibit NF-κB-induced inflammation as compared to the complete SOCS1 protein (Nakagawa et al., 2002; Ryo et al., 2003; Strebovsky et al., 2011; Zimmer et al., 2016). SOCS1 mRNA is naturally regulated through microRNA-155 at the post-transcriptional level (Yao et al., 2012), while post-translational regulation of SOCS1 includes phosphorylation by kinases like v-abl, pim1, and pim2. These kinases prevent the SOCS Box from binding to Elongin C, an important intermediate for E3 complex recruitment (Chen et al., 2002; Limnander et al., 2004). SOCS1 was discovered by three different groups simultaneously in the year 1997 led by Tadamitsu Kishimoto at the Osaka University Medical School, (Naka et al., 1997) Akihiko Yoshimura at the Institute of Life Sciences in Karume, (Endo et al., 1997) and by Douglas Hilton at the Walter and Elisa Hall Institute in Melbourne (Starr et al., 1997). *SOCS1* expression can be induced by a number of signaling molecules including IL-2, 4, 7, 10, 15, type I and II IFNs, TNFα, and Colony stimulating factors (CSFs) (Sakamoto et al., 1998; Sporri, 2001; Federici et al., 2002; Cornish et al., 2003; Ding et al., 2003; van de Geijn et al., 2004). The *SOCS1* gene is located on Chromosome 16.

Suppressor of cytokine signaling 1 not only modulates JAK/STAT pathways, but it can also regulate TLR signaling. TLRs are pattern recognition receptors that can identify conserved microbial molecules and upregulate immune response against them (Mogensen, 2009). SOCS1 regulates these responses by targeting intracellular signal transduction elements MAL (MyD88-adaptor-like protein / TIRAP), IRAK1 (IL-1 receptor-associated kinase), TRAF6 (TNF receptor-associated factor 6), and p65 (a subunit of NF-κB) for ubiquitin-mediated proteasomal degradation and can bind IRAK1 to modulate TLR4 responses. SOCS1 is also induced in a feedback mechanism followed by TLR activation and STAT1 signaling (Nakagawa et al., 2002; Mansell et al., 2006; Jager et al., 2011; Strebovsky et al., 2011; Zhou et al., 2015). A recent report has elucidated that the mechanism of SOCS1-mediated inhibition of kinase activity of JAK1, JAK2, and TYK2 is through binding to the GQM motif on the αG helix of the three above-mentioned kinases (Liau et al., 2018).

Suppressor of cytokine signaling 1 can regulate responses of type I IFN, which function through IFNAR1/2 and TYK2/JAK1; and type II IFN (IFN γ), which functions through IFNGR1/IFNGR2 and JAK1/JAK2 (Federici et al., 2002; Platanias, 2005). Additionally, SOCS1 modulates IL-12 signaling, gp130 (CD130) utilizing cytokines such as IL-6 and LIF, and common γ chain (CD132) utilizing cytokines such as IL-2 and IL-21 (Losman et al., 1999; Sporri, 2001; Eyles et al., 2002). Since SOCS1 has a profound role in T cell homeostasis, it is a prominent player in both autoimmunity and cancer. SOCS1^-/-^ mice die of perinatal autoinflammatory disease or lymphoid deficiencies, develop polycystic kidneys, and inflammatory lesions. While these mice can be partially saved by *IFNγ* deletion, these mice still develop fatal inflammatory diseases later (Starr et al., 1998; Alexander et al., 1999; Metcalf et al., 2002; Collins et al., 2011). *SOCS1* deficiency or dysregulated JAK/STAT signaling has been correlated with a number of immune disorders in humans, including SLE, scleritis, and asthma (Lee et al., 2009; Wang et al., 2010; Yu et al., 2011; Sukka-Ganesh and Larkin, 2016). *SOCS1^-/-^* Dendritic cells have an increased sensitivity to LPS and can often result in system autoimmune diseases (Hanada et al., 2003). Moreover, *SOCS1^-/-^* peripheral T cells show increased responsiveness to IL-2 and tend to have a skewed ratio of CD4/CD8 population (Cornish et al., 2003; Ilangumaran et al., 2003a,b).

A novel approach to combat *SOCS1* deficiency is the use of SOCS1 mimetics. A SOCS1 mimetic peptide containing only the n-terminal kinase inhibitory region (KIR 53- DTHFRTFRSHSDYRRI-68) domain has gained attention due to its effectiveness in JAK1/2 and TYK2 inhibition activity (Waiboci et al., 2007). The KIR domain binds to the activation loop of JAK1/2 and TYK2 to prevent them from phosphorylating their targets. It is an intrinsically disordered protein (IDP), lacking a tertiary structure prior to substrate engagement (Jirgensons, 1966; Uversky et al., 2000, 2005). It has been shown using circular dichroism that SOCS1 mimetic peptide can take up an α-helical structure upon addition of trifluoroethanol which highlights the peptide’s propensity to form stable secondary structure, allowing it to carry out its function (Recio et al., 2014). Of note, SOCS1^-/-^ mice have prolonged survival after treatment with this SOCS1 mimetic peptide (Collins et al., 2011). This approach has also been proven to have a beneficial effect in animal models of inflammatory diseases like EAE and Uveitis (Jager et al., 2011; He et al., 2016).

In this review, we highlight the importance of SOCS1 as a regulator of immune responses contributing to autoimmunity/autoinflammation and cancer and the potential use of SOCS1 mimetic peptide or gene therapy as treatment tactic.

### Psoriasis

Psoriasis Vulgaris (PsO) is a dermatological disease marked by plaques and erythematosus on the skin. Histological analyses reveal excess keratinocyte proliferation (acanthosis) and lymphocyte infiltration into the epidermis (Griffiths and Barker, 2007; Menter et al., 2008; Chiricozzi et al., 2011; Guttman-Yassky et al., 2011; Lin et al., 2011). Five types of PsO exist – (1) Plaque-type: most common form of psoriasis and manifests as marked erythematous plaques and squamous lesions localized on elbows, scalp, knees, and sacral zone; (2) Inverse type: plaques localized to skin folds but squamous lesions do not form; (3) Glutate type: frequent in children and adolescents, lesions appear like small droplets and often manifest after a streptococcal infection; (4) Pustular type: rarely occurs and is marked by the presence of pustules on the skin; (5) Erythrodermic: lesions affect about 80% of the body surface accompanied by widespread vasodilation (Koca, 2016). The national psoriasis foundation (NPF) defines three levels of severity – mild psoriatic skin (<3% of body surface covered), moderate psoriatic skin (3–10% body surface covered, and severe psoriatic skin (>10% body surface affected) (Pariser et al., 2007). PASI (Psoriasis area severity index; a measure of average redness, thickness, and scaliness of the lesions) and PGA (Physician global assessment; based on a single estimate to represent the patient’s disease severity as assigned by the physician) are other classifications used to define the disease severity (Langley and Ellis, 2004; Feldman, 2005). There are multiple genetic susceptibility loci known including – *AIM2* [dsDNA cytosolic receptor aim2], *IL1RL1* [Interleukin 1 receptor-like 1], *IFNGR2* [Interferon γ receptor 2], *IL12B* [interleukin 12β], *TNIP1* [TNFAIP3-interacting protein 1], *TNFAIP3* [TNFα induced protein 3], and *NFKBIA* (NF-κB inhibitor A) (Loft et al., 2018; Tejasvi et al., 2012; Zuo et al., 2015). Even though the etiology is not clearly known, PsO can begin with bacterial infection followed by the release of anti-microbial peptides (Weisenseel, 2002). In a study by Munz et al. (2010), 16S rRNA sequencing of blood samples from 20 patients of psoriasis shed light on the presence of *Staphylococci* and/or *Streptococci*, depending on the type of psoriasis, suggesting an association between psoriasis and bacteremia (Munz et al., 2010). Certain anti-microbial peptides – LL37 and β-Defensins have been vastly implicated in the pathogenesis of psoriasis. LL37 complexes with host self-nucleic acids and ligate dendritic cells via TLR7 and TLR9, leading to loss of self-nucleic acid tolerance (Lande et al., 2007; Hollox et al., 2008; Ganguly et al., 2009). LL37 can protect keratinocytes from apoptosis, further aiding to psoriasis phenotype. LL37 and keratin-7 are some of the autoantigens targeted by T lymphocytes in psoriasis. Previously thought to be Th1-dominant disease, it is now known to be both Th1 and Th17 driven disease (Lee et al., 2004; Blauvelt, 2007). Since SOCS1 is a classical regulator of IFN-γ and IL-6 signaling, SOCS1 can skew T cells responses away from Th1 and Th17 (Starr et al., 1997; Alexander et al., 1999; Diehl et al., 2000). Interestingly, Foxp3^+^ regulatory T cells tend to show increased plasticity and lean toward Th1/Th17 phenotype when deficient in SOCS1 (Collins et al., 2011; Takahashi et al., 2011, 2017).

Psoriasis-like lesions can be induced on mouse skin by topical application of imiquimod, a TLR7 agonist (van der Fits et al., 2009; Lande et al., 2014). The imiquimod-induced mouse model for psoriasis shows similar histological and phenotypical characteristics to human plaque psoriasis and is believed to be a reliable induced model for studying the human disease (Palamara et al., 2004; van der Fits et al., 2009). TLR7/8 signaling in pDCs (Plasmacytoid dendritic cells) and MoDCs, in synergy with TLR4 signaling, can stimulate expression of IL12p35, IL23p19, and IL-6 which mediate Th1/Th17 polarization resulting in secretion of IFN-γ or IL-17 by Th1/NK and Th17 cells, respectively (Mosmann and Sad, 1996; Hamalainen et al., 2001; Acosta-Rodriguez et al., 2007; Nakae et al., 2007; Xu et al., 2007). IFN-γ is a potent activator of macrophages and inducer of CXCL9 (MIG) and CXCL10 (IP-10) in the epidermis, which then aids to recruit CXCR3^+^ Th1 cells, NK cells, and neutrophils to aggravate inflammation during early stages of the disease (Klunker et al., 2003; Ottaviani et al., 2006; Ferrari et al., 2015). It should be noted that CXCL10 is a strong biomarker of psoriasis, found in psoriatic plaques, and an active therapeutic target (Gottlieb, 1988; Ferrari et al., 2015). In a similar manner, TLR7 agonists can induce CCL2 (MCP-1) secretion by macrophages which then recruits CCR2^+^ Th17 cells and monocytes to the epidermis (Lembo et al., 2014). SOCS1 also maintains the expression of CCR7 on naïve T lymphocytes and aids in retaining them in the secondary lymphoid organs, highlighting a key role of SOCS1 is preventing unwarranted infiltration of naïve T cells into peripheral tissues like the skin (Yu et al., 2008). IL-17 can signal in both hematopoietic and non-hematopoietic cells. Apart from its regular housekeeping functions and synergizing with TNF-α, IL-17 can act as an amplifier of inflammation as it can stabilize other cytokines’ mRNA by inducing expression of RNA stabilizing intracellular protein *HuR* (Amatya et al., 2017). TLR7 signaling is particularly amplified in pDCs due to their relatively high expression of TLR7 (Jarrossay et al., 2001; Kadowaki et al., 2001; Hänsel et al., 2011). Furthermore, TLR7 and 8 signaling have been known to upregulate their own gene expression, in an autocrine fashion (Lombardi et al., 2009). Of note, a recent study by Yu et al. (2018) has demonstrated TLR7 signaling also induces *SOCS1* gene expression and that SOCS1 can suppress TLR7-mediated type-1 IFN secretion by pDCs, another vital element in psoriasis pathogenesis. The mechanism is both through IRF7 degradation, required for TLR7 signaling, and the inhibition of tyk2, required for type-1 IFN signaling (Gilliet et al., 2008; Piganis et al., 2011; Baldwin et al., 2013; Gui et al., 2016; Yu et al., 2018). UV-B narrow band can also reduce type-1 IFN signaling via facilitating phosphorylation-dependent ubiquitination of the IFN receptor chain – IFNAR1 (Gui et al., 2016). Grine et al. (2015) reported that IFNAR1-deficient mice were partially protected from Imiquimod-induced inflammation.

Th17 cells are major players in imiquimod-induced psoriasis as well, and pDCs have been reported to secrete pro-Th17 cytokines (e.g., IL-6) in response to TLR7 ligation (Yu et al., 2010). Kim et al. (2016) showed that upon imiquimod application, the CD27-Vγ1- γδ T cell population was significantly increased. Moreover, anti-p40 (a subunit shared by IL-12 and IL-23) and PD-L1-F fusion protein therapy resulted in assuagement of the disease (Krueger et al., 2007; Kim et al., 2016). Moreover, IL-22, a Th17 cytokine, has been implicated in promoting acanthosis and immune cell recruitment in the epidermis and high serum levels of IL-22 are correlated with disease severity (Boniface et al., 2005; Wolk et al., 2006). IL-17A, the first member of the IL-17 family, has a distinct pattern of gene regulation in differentiated and undifferentiated keratinocytes. Spleen tyrosine kinase (Syk) can mediate IL-17 induced gene expression in keratinocytes and is also involved in TLR7 signaling, making it an interesting candidate to study in the context of psoriasis (Chiricozzi et al., 2014; Wu et al., 2015; Aouar et al., 2016). In a Syk-independent branch of the pathway, IL-17 signaling has a unique ability to synergize with other cytokine signaling pathways by stabilizing their downstream gene transcripts and preventing their degradation, leading to an amplified inflammatory response (Amatya et al., 2017). Anti-IL-17A MAb therapy has been approved for treatment of moderate to severe psoriasis plaques, though some patients with moderate to severe psoriasis plaques have been known to suffer from unexpected side effects like nasopharyngitis, arthralgia, and upper respiratory tract infections (Papp et al., 2013; Rich et al., 2013; Langley et al., 2014; Gordon et al., 2016). Moreover, the application of anti-IL-17A therapy in patients with mild psoriasis is limited in the context of risk versus benefit as IL-17 plays a protective and reparative role in the gut and barrier tissues (Song et al., 2015). As such, therapeutic alternatives to anti-IL-17 therapies remain an unmet need in individuals with mild to moderate disease.

**FIGURE 3:**
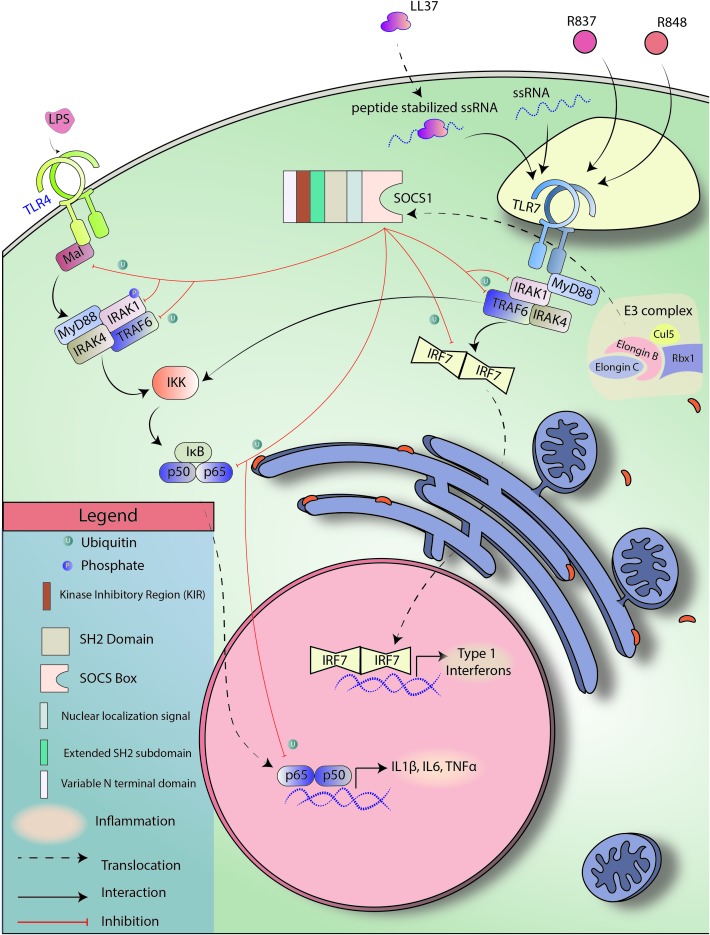
SOCS1 regulates TLR signaling. TLR4 and TLR7 are pattern recognition receptors known to be responsive toward LPS and ssRNA, respectively. TLR4 and TLR7 signal through NF-kB and IRF7, respectively to induce the expression of target genes. SOCS1 can induce SOCS Box-mediated ubiquitination of Mal, TRAF6, p65, and IRF7 to block both the signaling cascades.

TLR4 expression is upregulated in PBMCs in human patients with psoriasis, and variants of TLR4 are implicated in both plaque-type psoriasis and psoriatic arthritis (Garcia-Rodriguez et al., 2013; Panzer et al., 2014; Smith et al., 2016). TLR4 can interact with bacteria endotoxin LPS and initiate an inflammatory signaling cascade (Janssens and Beyaert, 2003). SOCS1 can regulate TLR4 mediated inflammation by inducing degradation of TRAF6, IRAK1, and Mal protein, which subsequently prevents p65 phosphorylation and activation (Nakagawa et al., 2002; Mansell et al., 2006; Jager et al., 2011; Zhou et al., 2015). The importance of SOCS1 is highlighted in that SOCS1 knockout macrophages have increased sensitivity to LPS (Sachithanandan et al., 2011). TLR4 signaling may also mediate crosstalk with STAT3 signaling via MyD88-induced IL-6 (Yamawaki et al., 2010). STAT3 is a widely accepted oncogene and inflammatory mediator that will be discussed later in this article. STAT3 is an interesting target in the context of psoriasis as it is required for IL-6, IL-22, and IL-23 signaling and has been linked with the development of psoriasis in a transgenic mouse model (Sano et al., 2005; Figure 3 touches upon SOCS1-mediated TLR regulation briefly).

### Cancer

Anomalous gene expression by cancer cells can lead to cell transformations. Such transformed cells can sustain unimpeded growth, evade contact inhibition, ignore apoptotic signals, undergo metastasis and angiogenesis, and evade the immune response (Hanahan and Weinberg, 2011). While the JAK/STAT pathway is required for cytokine signaling and alerting the immune system for tumor clearance, it can also facilitate tissue survival and neoplasia (Classen et al., 2009; Bunker et al., 2015; La Fortezza et al., 2016). SOCS-1, being a classical regulator of JAK/STAT signaling, is a potent tumor suppressor as aberrant SOCS1 gene methylation and allelic mutations have been linked to different types of malignant cancers (Fujitake et al., 2004; Melzner, 2005, Melzner et al., 2006). Epigenetic inactivation due to CpG methylation of SOCS1 is frequently linked to Hepatocellular carcinoma, human gastric carcinoma, melanoma, multiple myeloma, pancreatic ductal neoplasm, and acute myeloid leukemia (Franke, 2001; Yoshikawa et al., 2001; Chen et al., 2003; Fukushima et al., 2003; Galm, 2003; Oshimo et al., 2004; Mottok et al., 2007; Liu S. et al., 2008). SOCS1 mediated negative feedback signaling is paramount for not only reducing inflammation, but also to curb unchecked cell growth.

**FIGURE 4:**
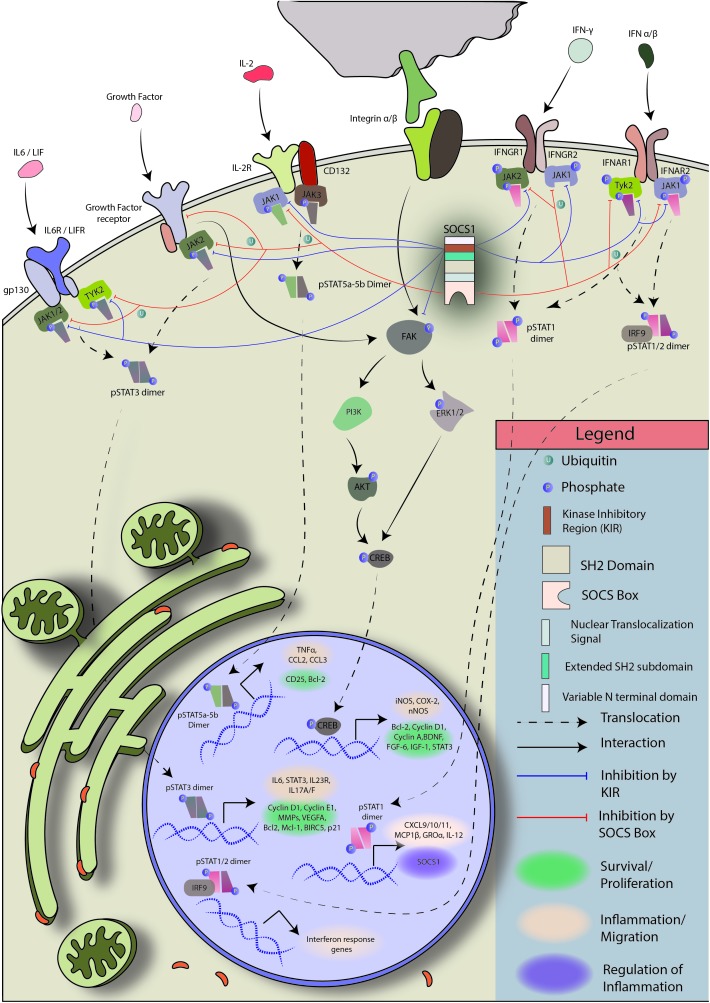
Regulation of JAK/STAT and FAK signaling by SOCS1: Janus kinases (JAKs) are physically close to cytokine receptors. When a cytokine binds its cognate receptor, the respective JAK phosphorylates itself and the cytokine receptor. Receptor phosphorylation creates docking sites for STAT protein binding and brings the associated JAKs in propinquity. JAK autophosphorylation is required for enzymatic activation. The activated JAKs then phosphorylate their target pre-formed STAT dimers or STAT monomers, which form homo/heterodimers and enter the nucleus to initiate transcription. SOCS1 is a regulator of JAK1/2 and TYK2. It can block phosphorylated JAK1, JAK2, and TYK2 to prevent STAT activation and dimer formation, putting a halt to JAK/STAT signal transduction (Braunstein et al., 2003; McNally and Eck, 2014).

Suppressor of cytokine signaling 1 has been shown to regulate, directly or indirectly, a number of molecules and pathways that have been implicated in cancer – CDK2, CDK4, Cyclin D1, MAPK/p38, PDL1, STAT1, STAT3, STAT6, p53, p21, FAK, E-cadherins, Met tyrosine kinase, type I and II IFN, and numerous proinflammatory cytokines (Liu, 2003; Ritz et al., 2008; Neuwirt et al., 2009; Souma et al., 2012; David et al., 2014; Gui et al., 2015; Yeganeh et al., 2016; Liau et al., 2018; Figure 4 briefly explains the mode of regulation by SOCS1).

One of the ways non-immune cells, including cancer cells, promote immune-suppression and induction of tolerance is by expressing PDL1 (also known as B7.H1 or CD274), a ligand for PD1 expressed by T cells. The interaction between PD1 and PDL1 can cause anergy or even programmed cell death in T cells (Dong et al., 2002). Anti-PDL1 is a common anti-cancer therapy facilitating immune cells recognition and obliteration of tumor cells. IFN β and γ receptor signaling contribute to significantly increase the expression of PDL1 through the JAK1/2-STAT1/2/3-IRF1 axis (Garcia-Diaz et al., 2017). Since SOCS1 is a natural regulator of both type-I and II IFN signaling and JAK1/2/STAT1 signaling, it also indirectly regulates PDL1 expression. Nonetheless, there are also contradicting reports that suggest increased *SOCS1* expression as an accomplice in melanoma, colorectal cancer, breast cancer, and neuroendocrine cancer (Raccurt et al., 2003; Li et al., 2004; Laner-Plamberger et al., 2013; Tobelaim et al., 2015; Berzaghi et al., 2017). Hence, further studies are required to elucidate alternative pathways modulated by SOCS1 and cell-type specific functions. It should also be noted that *SOCS1* overexpression has been reported to subvert IFN-α therapy in chronic myeloid leukemia, therefore, a balance, rather than an excess, of SOCS1 is required for normal cell functioning.

STAT3 (also known as acute phase response factor) is DNA-binding, an intracellular signaling protein that has pleiotropic effects on embryogenesis, oncogenesis, tumor suppression, cell differentiation, growth, and both innate and adaptive immunity (Akira, 2000; de la Iglesia et al., 2008, 2009). IL-6 signaling is known to induce *STAT3* gene expression and its phosphorylation-mediated activation resulting in the transcription of STAT3 target genes (Ichiba et al., 1998). The activation of STAT3 can be induced by a variety of cytokines including IL-6 and IFN-α (Puthier et al., 1999). Intriguingly, unphosphorylated STAT3, in response to IL-6, has also been reported to aid in inflammation by interacting with NF-κB and upregulating *CCL5*, *IL8*, *IFNβ*, and *ICAM1* (Matikainen et al., 1999; Yang et al., 2007). In addition to non-receptor kinases such as JAKs, Bcr-abl1, and Src., STAT3 can also be phosphorylated by growth factor associated kinases, like Trk, (Ng et al., 2006; Al Zaid Siddiquee and Turkson, 2008). Under physiological conditions, STAT3 signaling is highly regulated. However, under pathological conditions activated STAT3 has been implicated in hematological and non-hematological tumors, largely through promotion of autocrine IL-6 signaling and secretion that drives cancer progression and multidrug resistance (Koudstaal et al., 1967; Buettner et al., 2002; Yu and Jove, 2004; Yeh et al., 2006; Al Zaid Siddiquee and Turkson, 2008; Huang et al., 2010; Mace et al., 2013; Cheng et al., 2016). Mouse fibroblasts artificially induced to have constitutive expression of dimerized pSTAT3 were autonomously capable of causing tumors when transferred to nude mice (Bromberg et al., 1999). Moreover, aberrant IL6/JAK/STAT3 signaling has been observed in human patients of cervical, breast, ovarian, head and neck, colorectal, prostate, renal, oesophageal, non-small cancers, brain cancers, sezary syndrome, retinoblastoma, and lymphoma (Eriksen et al., 2001; Rahaman et al., 2002; Zhang et al., 2002; Chung and Chang, 2003; Chang C.H. et al., 2013; Konnikova et al., 2003; Riedel et al., 2005; Lane et al., 2011; Lesina et al., 2011; Culig and Puhr, 2012; Chen et al., 2013; Jo et al., 2014; Jinno et al., 2015; Kotowicz et al., 2016; Kitamura et al., 2017; Johnson et al., 2018). STAT3 phosphorylation followed by irradiation therapy and chemotherapy presents a challenge for cancer treatment since pSTAT3 contributes to the transcription of anti-apoptotic genes including *Mcl1*, *Bcl2*, *Bcl-xL*, and *BIRC5* (Bromberg et al., 1999; Catlett-Falcone et al., 1999; Alas and Bonavida, 2001; Real et al., 2002; Diaz, 2006; Kujawski et al., 2008; Yu et al., 2013). Irradiated breast cancer cells have been reported to secrete SASP factors, including IL-6, which aides in tumor progression, angiogenesis, and metastasis (Kujawski et al., 2008; Barbieri et al., 2010; Yu et al., 2013). STAT3 has been known to induce *HIF-1α* gene expression, required for tumors to survive in hypoxia, and can also regulate p53, Cyclin D1, E1, and p21 (Kortylewski et al., 2005; Niu et al., 2005, 2008; Chang Q. et al., 2013). Furthermore, aberrant *STAT3* expression may play a role in maintaining survival and plasticity of cancer stem cells, as STAT3 is known to support pluripotency by upregulating *sox2* [SRY-box 2], *Nanog* [Homeobox protein nanog], and *c-myc* [MYC proto-onco gene] (Kiuchi et al., 1999; Foshay and Gallicano, 2008; Gregory et al., 2008; Kamiya et al., 2011). Constitutive pSTAT3 signaling is also known to provide resistance to chemotherapy in breast cancer cells via a similar downstream process as mentioned previously (Real et al., 2002). It is worth noting that STAT3 integrates with the PI3K pathway, another major signaling pathway governing cell survival and apoptosis, by regulating the expression of the regulatory subunits of the Class IA PI3K enzyme during lactation and involution (Abell and Watson, 2005). Various STAT3 inhibitors have been studied to inhibit cell proliferation in cancer cell lines (Swiatek-Machado et al., 2012). Moreover, STAT6, a typical Th2 signaling molecule, has been reported to play role in glioma progression as well, both alone, and as an accomplice to STAT3 (Merk et al., 2011; Yan et al., 2016). SOCS1 is one of the natural regulators of STAT3 and STAT6 signaling and has tremendous potential as therapeutic. SOCS1 can localize to the nucleus via NLS and aid in p53 phosphorylation, hence, it is also a part of DNA damage response (Mallette et al., 2010). This partially explains why SOCS1^-/-^ mice are relatively more susceptible to cancer. SOCS1 can also regulate many cell cycle components directly. Natatsuka et al. (2015) demonstrated that SOCS1 can bind ATR through p53 and cause a G2/M arrest of gastric cancer cell lines. Of note, SOCS1 adenoviral gene therapy has been shown to impede cell growth in gastric cancer cells by reducing levels of pSTAT3 (Natatsuka et al., 2015; Sugase et al., 2018). Similarly, SOCS1 gene therapy was also shown to augment irradiation mediated DNA damage in Esophageal Squamous Cell Carcinoma (ESCC) (Souma et al., 2012; Sugase et al., 2017). The twist in the story comes with STAT1, which is primarily associated as a target of SOCS1-meditated regulation. STAT1 is known to have both tumor suppressive and oncogenic effects (Rock et al., 2018). Aberrantly low *STAT1* expression is reported to have a poor clinical outcome in several cancers, including melanoma and breast cancer. *STAT1^-/-^*mice are more prone to experimentally induced tumors, as well as develop cancer spontaneously (Lesinski et al., 2003; Chan et al., 2012; Hosui et al., 2012; Hix et al., 2013). Activated STAT1 is also known to cooperate with p53 to induce apoptosis in malignant cancer cells (Forys et al., 2014; Youlyouz-Marfak et al., 2008). Part of the tumor suppressive effects can be ascribed to heterodimer formation with STAT3. The STAT1-STAT3 heterodimer governs transcription of a different set of genes, often resulting in apoptosis instead of survival (Thyrell et al., 2007; Regis et al., 2008). Moreover, STAT1 and STAT3 reciprocally regulate each other’s expression and activity and even compete for JAK docking sites for phosphorylation (You et al., 2013; Friedrich et al., 2017). On the other hand, several studies have portrayed *STAT1* masquerading as an oncogene, more of which is described in the following review (Rock et al., 2018). This scenario highlights the complexity of the effects of SOCS-mediated regulation.

*Focal Adhesion Kinases* (or protein tyrosine kinase 2) reside within focal adhesions where the cell cytoskeleton contacts the extracellular matrix. FAK activation can result from receptor signaling via receptor tyrosine kinases (RTK), cytokine receptors, growth factor receptors, G-protein coupled receptors, and integrins through FERM domain interactions (Frame et al., 2010). There is increasing evidence that FAK autophosphorylation at Y397 is associated with oncogenesis by aiding in cell migration, FAK activity can lead to turnover of focal adhesion points and upregulation of MMPs, and the inhibition of the autophosphorylation has been shown to reduce tumor growth (Sieg et al., 2000; Hauck, 2002; Dunty et al., 2004; Cui et al., 2006; Hochwald et al., 2009; Heffler et al., 2013). One of the reported mechanisms for focal adhesion turnover is through phosphorylation of actin-binding protein cortactin (Tomar et al., 2012). The blockade of FAK-Cortactin signaling pathway has been shown to make cells susceptible to radiation therapy in head and neck cancer (Eke et al., 2012). Furthermore, *MMP9* expression by FAK signaling is implicated in orthotopic breast cancer metastasis (Mitra et al., 2006). FAK governs not only tumors, but also stromal cell biology (Sulzmaier et al., 2014). Besides the traditional roles of FAK, it is also involved in epithelial-to-mesenchymal transition (EMT), bypassing apoptosis, and angiogenesis (Xu et al., 2000; Kurenova et al., 2004; McLean et al., 2005; Weis et al., 2008; Zouq et al., 2009; Canel et al., 2013; Fan et al., 2013). Activated FAK protein can interact with src-kinase to form a dual-kinase complex and then upregulate MAPK-ERK kinase cascade to induce migration (Schlaepfer and Hunter, 1997). αvβ5 integrin–FAK–AKT signaling pathway blockade has been reported to prevent attachment-dependent apoptosis in murine ovarian carcinoma cells (Lane et al., 2010). FAK pharmacological inhibitors are being investigated as cancer chemotherapeutics and FAK inhibition has been shown to ameliorate tumor growth, metastasis, and angiogenesis in mouse models of adenocarcinoma, ovarian carcinoma, pancreatic cancer, and non-small lung cancer (Halder et al., 2007; Slack-Davis et al., 2009; Lane et al., 2010; Stokes et al., 2011; Chen et al., 2012; Jean et al., 2014). SOCS1 is known to be induced by PDGF and integrin signaling. SOCS1 binds directly with Y397-phosphorylated FAK through the SH2 and KIR domains and induce ubiquitination followed by proteasomal degradation (Liu, 2003). SOCS1 also modulates JAK/STAT signaling of other growth factors which lie upstream of FAK in mice therefore indirectly regulating FAK signaling. Ergo, SOCS1 holds great potential in the treatment of FAK-driven cancers. Of note, a quite recent study showed that SOCS1 gene therapy can prohibit proliferation of gastrointestinal stromal tumors by interfering with FAK and PI3K pathway (Sugase et al., 2018). One of the challenges with FAK inhibition therapy would be the fact that PYK2, a homolog with similar function, can substitute for FAK activity in its absence, and hence, the inhibitors need to be carefully designed or a combinatorial therapy should be preferred. While there is some evidence that SOCS1 may become associated with PYK2 for activity modulation, there is a need to elucidate the mechanism and confirm the finding before drawing a strong conclusion (Masuhara et al., 1997). Another challenge would be to consider the pleiotropic functions of FAK in housekeeping tasks.

### Lupus

Like most autoimmune diseases, Lupus’ etiology is not clearly known. A well-established model for studying SLE is MRL/LPR mice, which are known to develop lupus-like pathology and clinical manifestation closely resembling the human condition, spontaneously (Perry et al., 2011). Splenomegaly and lymphadenopathy due to hyperproliferation of CD3^+^ CD4^-^ CD8^-^ T cells is an immunological feature of these mice (Zhang et al., 2009). Lupus-like pathology can also be created by topical treatment of mice with TLR7 agonists like R837 (imiquimod) and R848 (resiquimod) (Yokogawa et al., 2014). While TLR7 signaling exacerbates the disease condition, a recent study has found that TLR9 may have a protective role in SLE since *TLR9^-/-^* mice had an accelerated disease phenotype (Liu et al., 2018). Polymorphism in *SOCS1* gene has been correlated with the occurrence of SLE (Chan et al., 2010).

Malignant NETosis by neutrophils can lead to ANA production, one of the hallmarks of SLE (Yu and Su, 2013). These circulating ANA and nucleic acid can cause type I IFN secretion via TLR7/8 activation. Multiple reports have hinted toward the contribution of type I and II IFNs in disease priming and progression (Vallin et al., 1999; Baechler et al., 2003; Han et al., 2003; Hua et al., 2006; Lit, 2006; Elkon and Santer, 2012; Munroe et al., 2014). The ANA can induce more NETosis and perpetuate the cycle of inflammation (Murphy et al., 1998; Marzocchi-Machado et al., 2002; Lande et al., 2007, 2011; Garcia-Romo et al., 2011). The condition can be exacerbated by LL37-mediated stabilization of DNA (Lande et al., 2011). This anti-microbial peptide has also been known to induce M1-phenotype in macrophages and activating inflammasomes resulting in increased IL-18, another biomarker for SLE (Kahlenberg et al., 2011). TLR7-mediated self RNA ligation and duplication in the *TLR7* gene have been known to upregulate autoreactive B cell responses. Similar to psoriasis, LL37 can also complex with self-RNA, stabilize, and internalize it for TLR7 activation and promote inflammation and ANA production (Blanco et al., 1991; Pisitkun, 2006; Ganguly et al., 2009). Since the pro-inflammatory cytokines secreted in the abovementioned processes signal through the JAK/STAT pathway, the pathway becomes a particularly effective therapeutic target, especially in Lupus. It has also been reported that monocytes of SLE patients have hyperactive JAK/STAT signaling (Li et al., 2011).

Reduced expression of *SOCS1* and/or increased IFN-γ/IL-6 signaling are rampant in SLE rodent models and human patients (Baechler et al., 2003; Fujimoto, 2004; Harigai et al., 2008; Sharabi et al., 2009; Sukka-Ganesh and Larkin, 2016). Since SOCS1 is known to regulate JAK/STAT pathway and multiple TLR responses including TLR4 and TLR7, directly or indirectly, SOCS1 mimetics have a remarkable therapeutic potential which should be explored (Kinjyo et al., 2002; Nakagawa et al., 2002; Strebovsky et al., 2011; Yu et al., 2018). Recently, a small-molecule inhibitor of JAK1 and 3, tofacitinib, has been shown to assuage lupus progression in MRL/LPR mice (Clark et al., 2014; Furumoto et al., 2017).

The ANA, in complex with their target epitopes, make their way to the kidneys for clearance where they are phagocytosed by mesangial cells, leading to a condition called lupus nephritis (LN) which is a major cause of morbidity in SLE patients (Mak et al., 2007; Almaani et al., 2017). These phagocytosed antibodies can further create a nuisance by causing T cell infiltration into the mesangial membrane and induce upregulation of MHC II of the mesangial cells, leading to incessant inflammation and mesangial cell hypertrophy. Therefore, successful therapy for SLE should also clear these infiltrating leucocytes and reduce nephritis intensity. Our group has previously shown the importance of SOCS1 pathway in SLE and reduced SOCS1 expression in patients (Sukka-Ganesh and Larkin, 2016).

### Recurrent Uveitis

**FIGURE 5:**
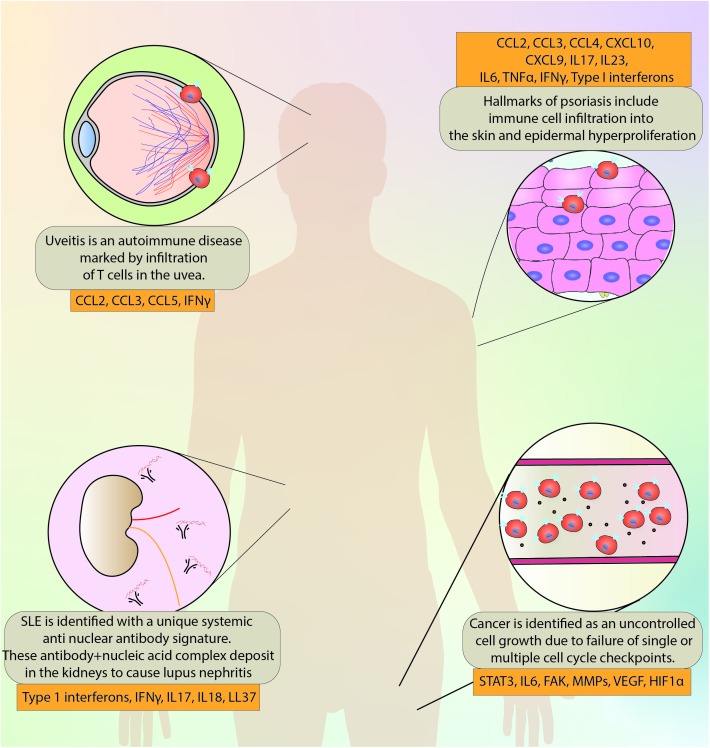
A brief overview of the disorders discussed in this review and the major molecules implicated in the pathogenesis and progression.

Uveitis is a severe disease of the eye that accounts for more than 10% of the visually handicapped population in the United States (Acharya et al., 2013; González et al., 2018). It can manifest as anterior uveitis, in the front of the eye, posterior uveitis, in the back of the eye, or pan uveitis, throughout the eye (Nussenblatt, 1990). The disease can be induced by immunizing with retinal antigens, using appropriate adjuvants (Wiechert et al., 2001; Deeg et al., 2002). The pathogenesis is mostly due to immune cells infiltrating the eye and causing inflammation, with pathologic T cells being the prominent drivers of the disease. Studies are carried out in mouse or rat model of EAU, however, the equine disease resembles the most to the human condition (Gilger et al., 1999; Wiechert et al., 2001; Deeg et al., 2004; Malalana et al., 2015). In equine recurrent uveitis, S-antigen and IRBP are the primary antigens but the polyclonal T cell expansion causes epitope spreading and hence brings out the recurrent nature of the disease. A similar pattern may be expected in human disease (Deeg et al., 2006). Migration of immune cells to the eye is an important event for the disease to progress. Chemokines containing the CC motif, including MIP-1α (CCL3), MCP-1 (CCL2), and RANTES (CCL5), have a prominent role in recruiting T cells and monocytes to the inflamed eye (Crane et al., 2001). *CCL2* can be upregulated by macrophages in response to IFN γ (Bauermeister, 1998), and RANTES expression is known to be modulated by STAT3 signaling (Yang et al., 2007). In addition, STAT3^-/-^ T cells were unable to mount a spontaneous autoimmune response. This highlights the prime role of JAKs in disease progression as both STAT3 and IFN-γ signaling depend on it for signal propagation. The importance of JAKs in the disease underscores the basis for using SOCS1 as a therapeutic approach (Liu X. et al., 2008). Current therapies include corticosteroids and NSAIDs, however, they have been known to cause severe side effects like glaucoma and cataract (Nussenblatt, 2002). Constitutive expression of *SOCS1* in the retina was reported to reduce recruitment of lymphocytes, resulting in reduced inflammation (Yu et al., 2011). Moreover, ocular topical treatment with a SOCS1 mimetic, containing the KIR region called SOCS1-KIR, has been shown to provide alleviation in the disease condition in Lewis rats and B10.RIII mice (He et al., 2015, 2016; Ahmed et al., 2018). As previously described, SOCS1 prevents naïve T cells from infiltrating ocular tissues by maintaining the expression of *CCR7*, required for retention of these cells in the secondary lymphoid organs where *CCL19 and CCL21* are constitutively expressed (Yu et al., 2008). SOCS1 is a therapeutic candidate worth exploring in the context of uveitis (see Figure 5 for summary).

### Other JAK Inhibitors–Approved and Under Investigation

Initial tests for the feasibility of tyrosine kinase inhibitors were done in the context of cancers in early 2000. Imatinib, a BCR-ABL tyrosine kinase inhibitor, was the first such inhibitor shown to be effective in chronic myelogenous leukemia (CML) and since then a number of kinase inhibitors have been approved by FDA for cancers (Druker et al., 2001). The success of these tyrosine kinase inhibitors undoubtedly paved a way for JAK inhibitors to undergo clinical trials. Ruxolitinib was the first FDA approved JAK inhibitor targeting tumors with hyperactive JAK/STAT signaling pathways (Harrison et al., 2012; Verstovsek et al., 2012). The approval of Ruxolitinib not only confirmed that JAK inhibitors were feasible but also safe for use as therapeutics. JAK inhibitors or jakinibs can regulate multiple cytokine and growth factor signals, while still allowing non-JAK signaling cytokines like TNFα, IL-1β, IL-17, and IL-8 to function normally, precluding an immunocompromised condition for the patient. Although, the same fact also underscores the limitation of this therapy, hence, jakinibs must not be confused for an autoimmunity panacea.

The first generation of jakinibs were pan-inhibitors, i.e., they blocked multiple JAKs. The shortcoming of the first generation of jakinibs is (1) they would also block JAKs required for anti-inflammatory cytokines, like IL-10, to signal (2) they could expose the patient to infections (3) they can block hematopoietic cytokines that typically signal through JAK1/JAK2 from functioning and cause cytopenia, which may not be desirable. Tofacitinib, a first generation JAK1/3 inhibitor, was the first jakinib to be approved for autoimmunity in humans (Meyer et al., 2010). It is a reversible competitive inhibitor for the ATP binding site of JAK1 and 3, and to a much less extent, of JAK2 and TYK2 (Philip et al., 1987). Initially, it was particularly advised for RA patients where methotrexate could not be administered or did not work, and later, it was proven to be non-inferior to the standard care – adalimumab, a TNF blocker (Fleischmann et al., 2017; Kotyla, 2018). Tofacitinib is also the most studied jakinib. There are other first-generation jakinibs in clinical trials for autoimmune diseases: Ruxolitinib (JAK1 and JAK2 inhibitor) for GVHD (NCT02913261), Psoriasis (NCT00617994), and Vitiligo (NCT02809976); Baricitinib (JAK1 and JAK2 inhibitor) for GVHD (NCT02759731) and Diabetic nephropathy (NCT01683409). In contrast, the second generation jakinibs are specific to a certain JAKs. This allows for a better targeting tactic with relatively reduced side effects than first gen jakinibs. Some of the second generation jakinibs being investigated are Upadacitinib (JAK1 inhibitor) for Atopic dermatitis and PF-06651600 (JAK3 inhibitor) for RA (NCT02969044) and ulcerative colitis (NCT02958865). Jakinibs have become a promising treatment for a range of immunity-related disorders including psoriasis, vitiligo, GVHD, lymphoma, solid tumors, SLE, ulcerative colitis, and atopic dermatitis (Buchert et al., 2016; Schwartz et al., 2017; Hosking et al., 2018).

Suppressor of cytokine signaling 1 KIR, a SOCS1 mimetic containing only the KIR domain, acts as a pseudosubstrate for JAK1, JAK2, and TYK2, with no known interaction with JAK3 (Liau et al., 2018). However, SOCS1 KIR can also interact with FAK, setting it apart from every other jakinib. What makes SOCS1 KIR more attractive as a therapeutic is its similarity to the naturally occurring protein SOCS1. Nonetheless, mimetic peptide drugs have disadvantages to their small molecule counterparts in terms of high cost, low permeability, proteolytic instability, and poor oral bioavailability, though a number of strategies are being used to improve these features in peptide drugs (Otvos and Wade, 2014; Di, 2015). For example, modifications like N-acetylation and c-amidation can improve peptide stability (Volonterio et al., 2003; Sato et al., 2006), use of lipid membrane and/or transporter systems for better bioavailability (Mahato et al., 2003; Griffin and O’Driscoll, 2011), and increasing serum protein binding of the peptide to reduce renal clearance (Pollaro and Heinis, 2010). Even though peptide drugs share an extremely small market share compared to small molecules (Vlieghe et al., 2010), their better specificity, low toxicity profile, and low drug-drug interaction potential makes them viable choice for the future once the challenges around their ADME (absorption, distribution, metabolism, and excretion) are overcome with progress in computational biology, metabolomics, and proteomics.

## Discussion

Suppressor of cytokine signaling 1 is an essential molecule for maintaining immune homeostasis and subverting inflammation. Disorders arising from excess inflammation or SOCS1 deficiency can be potentially treated with SOCS1 mimetics (Ahmed et al., 2015). While SOCS1 has promising potential in many disorders, it should be noted that new targets and actions of SOCS1 are still being discovered and not all the effects of this protein are beneficial in autoimmune diseases and cancer. For instance, SOCS1 degrades IRS1 and IRS2, required for insulin signaling, via the SOCS Box domain, thus, limiting its potential in type-2 diabetes (Rui et al., 2002). However, such challenges can be met by using SOCS1 mimetic peptide lacking the SOCS Box domain. As of now, SOCS1 gene therapy and mimetic-peptide biologics are active areas of research around the globe. Jakinibs have gained a great deal of attention in the last two decades for their efficacy in cancer and autoimmune diseases and we believe SOCS1 mimetics would be a great addition to the arsenal of jakinibs. Nonetheless, detailed safety and efficacy studies need to be carried before directly comparing SOCS1 mimetics to other jakinibs.

## Author Contributions

JS and JL wrote the manuscript. JS designed the figures. JL reviewed the figures.

## Conflict of Interest Statement

The authors declare that the research was conducted in the absence of any commercial or financial relationships that could be construed as a potential conflict of interest.

## Abbreviations

ANA: Antinuclear antibodies
ATR: Ataxia telangiectasia and Rad3 related
dsDNA: Double stranded deoxyribonucleic acid
EAE: Experimental autoimmune/allergic encephalomyelitis
EAU: Experimental autoimmune uveitis
ERU: Equine recurrent uveitis
FAK: Focal adhesion kinases
GVHD: Graft vs. Host Disease
IFN: Interferons
IL: Interleukin
IRBP: Interphotoreceptor retinoid-binding protein
IRF: Interferon regulatory factor
JAK: Janus Kinase
KIR: Kinase inhibitory region
MAb: Monoclonal antibody
MCP-1: Monocyte chemoattractant protein
MIP-1ββ: Macrophage inflammatory protein
MMP: Matrix Metalloproteinases
MoDCs: Monocyte-derived Dendritic Cells.
MS: Multiple sclerosis
NET: Neutrophil extracellular trap
NF κκB: Nuclear Factor κκB
NSAID: Non-steroidal anti-inflammatory drugs
PD1: Programmed cell death-1
PDL1: Programmed cell death ligand-1
pSTAT: phosphorylated Signal Transducer and Activator of Transcription
RANTES: Regulated on activation of normal T-cell-expressed and secreted
RNA: Ribonucleic acid
SASP: Senescence-associated secretory phenotype
SLE: Systemic lupus erythematosus
SOCS1: Suppressor of cytokine signaling 1
STAT: Signal transducer and activator of transcription
Th: T helper
TLR: Toll-like receptor
